# GILZ Regulates the Expression of Pro-Inflammatory Cytokines and Protects Against End-Organ Damage in a Model of Lupus

**DOI:** 10.3389/fimmu.2021.652800

**Published:** 2021-04-06

**Authors:** Champa Nataraja, Wendy Dankers, Jacqueline Flynn, Jacinta P. W. Lee, Wendy Zhu, Fabien B. Vincent, Linden J. Gearing, Joshua Ooi, Mehnaz Pervin, Megan A. Cristofaro, Rochelle Sherlock, Md Abul Hasnat, James Harris, Eric F. Morand, Sarah A. Jones

**Affiliations:** ^1^ Monash University Centre for Inflammatory Disease, School of Clinical Sciences at Monash Health, Melbourne, VIC, Australia; ^2^ Centre for Innate Immunity and Infectious Diseases, Department of Molecular and Translational Science, Hudson Institute, Melbourne, VIC, Australia

**Keywords:** GILZ, glucocorticoid, systemic lupus erythematosus, glomerulonephritis, IL-23

## Abstract

Glucocorticoid-induced leucine zipper (GILZ) mimics many of the anti-inflammatory effects of glucocorticoids, suggesting it as a point of therapeutic intervention that could bypass GC adverse effects. We previously reported that GILZ down-regulation is a feature of human SLE, and loss of GILZ permits the development of autoantibodies and lupus-like autoimmunity in mice. To further query the contribution of GILZ to protection against autoimmune inflammation, we studied the development of the lupus phenotype in Lyn-deficient (Lyn^-/-^) mice in which GILZ expression was genetically ablated. In Lyn^-/-^ mice, splenomegaly, glomerulonephritis, anti-dsDNA antibody titres and cytokine expression were exacerbated by GILZ deficiency, while other autoantibody titres and glomerular immune complex deposition were unaffected. Likewise, in patients with SLE, *GILZ* was inversely correlated with *IL23A*, and in SLE patients not taking glucocorticoids, *GILZ* was also inversely correlated with *BAFF* and *IL18*. This suggests that at the onset of autoimmunity, GILZ protects against tissue injury by modulating pro-inflammatory pathways, downstream of antibodies, to regulate the cycle of inflammation in SLE.

## Introduction

Glucocorticoid-induced leucine zipper (GILZ) exhibits a range of anti-inflammatory effects that invites significant interest as a potential target in developing an alternative therapeutic to glucocorticoids. Glucocorticoids continue to be used in the treatment of autoimmune diseases, particularly systemic lupus erythematosus (SLE), despite their predictable and severe adverse effects, because no safe and effective alternative has emerged. Glucocorticoids potently up-regulate GILZ, and thus understanding the potential for GILZ to protect against inflammation in SLE and other inflammatory conditions is imperative.

GILZ exerts anti-inflammatory actions by interacting with transcription factors to modulate important inflammatory signaling pathways, such as the NF-κB pathway, similar to the effects of glucocorticoids (1). As a result of its anti-inflammatory activity, GILZ is protective against damage in neuroinflammation (2), vascular, intestinal and liver inflammation (3–5), allergy (6), heart disease (7, 8), acute kidney injury (9), arthritis (10), psoriasis (11) and SLE. In SLE patients, active disease is associated with impaired GILZ expression (12). We previously showed that loss of GILZ exacerbates inflammation, exemplified by the development of a lupus-like phenotype in GILZ-deficient mice, characterized by antinuclear antibodies (ANA) and nephritis (12). Importantly, we have previously shown that GILZ is a non-redundant regulator of B cell activity, with GILZ deficiency resulting in heightened B cell activation and proliferation which was reversed by treatment with a cell-permeable GILZ fusion protein (12). These findings have important clinical correlates, for example, GILZ induction by glucocorticoids in most B cell subsets was negatively correlated with SLE disease activity (12). Additionally, others have independently confirmed that GILZ mRNA expression is negatively correlated with the SLE disease activity index (SLEDAI) (13, 14).

To gain further insights into the means by which GILZ protects against development of autoimmunity, we bred GILZ deficiency onto the Lyn knockout mouse model of lupus. Lyn is a member of the SRC family of protein tyrosine kinases that is a key negative regulator of signal transduction pathways in B cells, myeloid cells and dendritic cells (DC) (15, 16). Lyn phosphorylates inhibitory receptors in B cells that contain immunoreceptor tyrosine-based inhibitory motifs (ITIM) which initiate signaling events such as cytokine production, proliferation and migration (17). Thus, Lyn acts as a negative regulator of B cell activation, and consequently Lyn-deficient B cells are hyper-responsive to BCR ligand/signaling, with a lowered threshold to stimulation (18). Notably, Lyn-deficient mice spontaneously develop a well-characterized lupus-like autoimmune disease as they age that includes the loss of normal GILZ expression (12) and splenomegaly, autoantibody production, and severe immune complex-mediated glomerulonephritis (GN) (18, 19). Furthermore, pro-inflammatory cytokines such as BAFF, IL-6 and IFNγ play an important role in this phenotype, similar to human SLE (16, 20).

By examining the effect of GILZ deficiency on the Lyn^-/-^ model of lupus, we identified that, while loss of GILZ did not further exacerbate autoantibody expression, it did increase early damage to spleen and kidneys. This was accompanied by the appearance of pro-inflammatory cytokines at an earlier age in Lyn^-/-^ mice lacking GILZ than in those with sufficient GILZ. Thus, as appears to occur in human SLE, GILZ functions to limit the development of the inflammatory environment that contributes to end-organ damage.

## Materials and Methods

### Animals

The generation of GILZ-deficient mice has been previously described (21, 22), as has the generation of Lyn-deficient C57Bl/6 mice (18, 20). All GILZ-deficient mice are male, since GILZ is X-linked and renders male mice sterile (22) and female GILZ-deficient mice cannot be bred. Mice were housed in specific pathogen-free conditions. We generated GILZ-deficient mice on a Lyn-deficient background (GILZ/Lyn^-/-^) and compared them to wildtype (WT), GILZ knockout (GILZ^-/-^), and Lyn knockout (Lyn^-/-^) mice. WT and GILZ^-/-^ mice were co-housed as littermate controls, and Lyn^-/-^ and GILZ/Lyn^-/-^ animals were co-housed as littermate controls. All animals were housed in identical conditions in adjacent cages within our facility. Animals in experimental groups were carefully age and sex matched, but with ages pooled into <150 days or >200 days old so as to adequately power our statistical analyses. All procedures were approved by the Monash Medical Centre Animal Ethics Committee.

### Spleen Weights

**Figure 1:**
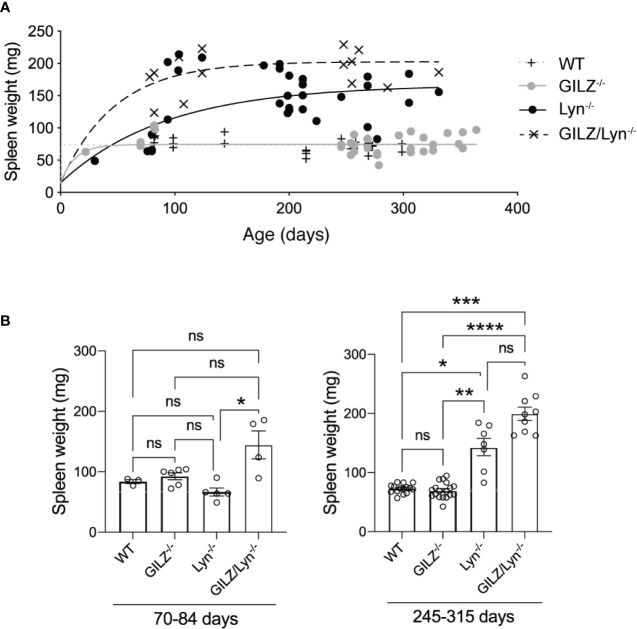
Effect of GILZ deficiency on development of splenomegaly in Lyn*-*deficient mice. **(A)** Spleen weights (mg) in male littermate mice according to age (days). Curves were fit to a one-phase decay model with robust regression. **(B)** Spleen weights in mice dichotomized according to the two age ranges 70-84 days (10-12 weeks) and 245-315 days (35-45 weeks). Kruskal-Wallis test followed by Dunn’s correction for multiple comparisons results are shown. *P<0.05, **P<0.01, ***P<0.005, ****P<0.0001. NS, not significant.

Mouse spleens were harvested and spleen weights were measured in milligrams (mg). Curves were fit to a one-phase decay model with robust regression using GraphPad Prism Software 7.0b. The age spans of mice chosen to display in **Figure 1B** were 70-84 days (10-12 weeks) and 245-315 days (35-45 weeks) so as to ensure adequate sample size and no significant differences in age that may alter the interpretation of the results. The statistics for the age groups are shown in **Table 1**.

**Table 1:** Number, mean and standard deviation of ages of mice included in the analysis shown in **Figure 1B**.

	70-84 days			245-315 days		
	*n*	*mean*	*SD*	*n*	*mean*	*SD*
**WT**	3	82.13	0.404	15	269.7	14.26
**GILZ^-/-^**	6	78.05	6.24	16	275.1	19.65
**Lyn^-/-^**	4	78.85	1.90	7	277.1	21.29
**GILZ/Lyn^-/-^**	4	80.93	1.95	9	239.6	39.15

A Kruskal Wallis test followed by Dunn’s correction for multiple comparisons found no significant differences between any age groups in the 70-84 day old cohort. In the 245-315 day cohort, there was a significant difference, (GILZ^-/-^ mice significantly were older than GILZ/Lyn^-/-^ mice, *P*=0.03), and no other significant differences were detected between ages of mice across genotypes.

### Cytokine Measurements

Serum was isolated from whole blood obtained by cardiac puncture following CO_2_ asphyxiation. Concentrations of BAFF, IFNγ, IL-10, IL-1α, IL-1β, IL-18, IL-12p70, IL-6, IL-23A and IL-17A, were measured in serum by Luminex as described in the manufacturer’s protocol (Customized mouse 11-plex ProcartaPlex Kit, Invitrogen).

### Autoantibody Measurement

Serum samples were used at 1:200 dilution for the identification of antibodies to extractable nuclear antigens (ENA) by flow cytometry. The FIDIS Connective Profile MX 117 kit (Theradiag) was used as per the manufacturer’s instructions with the variation of the use of a PE-conjugated anti-mouse IgG F(ab’)2 secondary antibody (eBioscience) for flow cytometry (23).

### Histology and Immunofluorescence of Kidneys

Kidneys from 8 to 45-week-old male mice were frozen in Optimal Cutting Temperature (OCT) compound (Tissue Tek). Frozen sections were stained with PE-conjugated polyclonal anti-mouse IgG F(ab’)2 secondary antibody (eBioscience) and FITC-conjugated goat IgG fraction to detect mouse complement component C3 (MP Biomedicals). Formalin fixed sections were stained with Haematoxylin and Eosin or periodic acid-Schiff (PAS) stain. Glomerular segmental necrosis was defined as acellular areas of PAS positive staining (24).

Glomerular damage was scored in a blinded manner for segmental necrosis (shown as percentage of glomeruli affected) and crescent formation as described (25). IgG and C3 immunofluorescence staining were given an intensity score between 0–3, and an average of 20 glomeruli were scored for each mouse.

### Measurement of Gene Expression

To assess GILZ expression in healthy subjects, we mined the publicly available datasets GSE123698 and GSE69832 for the probes corresponding to GILZ (gene name *TSC22D3*), as shown in **Supplemental Figure 2** (26, 27). To measure gene expression in patients with SLE, we mined the dataset GSE88884, which contains gene expression data from PBMC from n = 60 healthy subjects and n = 1,760 SLE patients enrolled in the ILLUMINATE-1 and ILLUMINATE-2 tabalumab phase III clinical trials (28, 29), taken at baseline. We extracted the data for the probes TC0X001262.hg.1:1, TC0X001262.hg.1:2 and TC0X001262.hg.1:3 that identified GILZ (gene name *TSC22D3*). Relative expression values for GILZ were determined by averaging the three probe set values for each subject, without batch correction, as with previous analyses. The strength of associations between GILZ and cytokine expression were determined based on Spearman’s rank order correlation. Clinical data were kindly provided by Dr Robert Hoffman of Eli Lilly.

### Statistical Analysis

All analyses and data visualization were performed using GraphPad Prism Software 7.0b and R software (version 4.0.2). Continuous and categorical data are presented as mean (standard deviation (SD)) or median (interquartile range) (IQR) and frequencies (percentage), respectively, according to data distribution. Choice of parametric/non-parametric test was guided by the assessment of continuous data distribution, as examined by Shapiro-Wilk and Kolmogorov-Smirnov tests. Correlation between variables was analyzed using Spearman’s rank correlation test. Difference in continuous variable between 2 or more than 2 groups were examined using Student’s unpaired two-tailed *t*-test with Welch’s correction, or Kruskal-Wallis test followed by Dunn’s multiple comparison test, respectively, where appropriate. A *P* value of < 0.05 was deemed statistically significant.

## Results

### GILZ Deficiency Increased Spleen Weight in Lyn-Deficient Mice

Splenomegaly occurs in some patients with active SLE and has also been described in both Lyn^-/-^ and GILZ^-/-^ mice (30). In Lyn*^-/-^* mice, splenomegaly is driven by the accumulation of plasma cells and Mac-1^+^ lymphoblasts, from as early as 12 weeks of age (31). GILZ-deficient mice develop splenomegaly as they age and isotype-switched plasma cells in spleen are increased (12). We therefore examined whether Lyn and GILZ synergized to regulate the processes that cause splenomegaly. We observed that Lyn-deficient mice developed splenomegaly at an early age, more so when GILZ was deficient, and spleen size progressively increased with aging (**Figures 1A, B**). While a Kruskal-Wallis test followed by Dunn’s correction for multiple comparisons did not detect a statistically significant difference between Lyn^-/-^ and GILZ/Lyn^-/-^ mice of 35-45 weeks of age when all genotypes were included in the analysis (**Figure 1B**), a Student’s t-test between these two genotypes showed a significant difference (*P*=0.0077). We did not observe splenomegaly in GILZ-deficient mice (**Figures 1A, B**), in contrast to our previous report (12), which is likely due to a change in housing conditions between these studies, as microbiota and cohousing may affect the phenotypes (32). A detailed analysis of the microorganisms detected within sentinel animals in the same room in which our colonies were housed in shown in **Supplemental Figure 1**, although we did not test whether the changes impacted on the development of an autoimmune phenotype in the GILZ^-/-^ strain. However, splenomegaly observed in Lyn-deficient mice was hastened in the absence of GILZ, and was worsened over time (**Figures 1A, B**). This detailed kinetic study of the degree of splenomegaly confirms that GILZ deficiency is permissive of the development of autoimmunity triggered by underlying genetic factors.

GILZ has recently been reported to decrease in murine macrophages as a function of the mild inflammatory state that develops with age (33). We previously showed that Lyn^-/-^ mice expressed lower GILZ, like patients with SLE, and this deficiency worsened with age (12). In the Lyn^-/-^ model, the development of autoimmunity is exacerbated over time and severity increases with age (34). However, in wildtype mice (12), and in peripheral blood mononuclear cells from healthy subjects (26, 27) and patients with SLE (35), we detected no meaningful change in GILZ expression that occurred over the lifespan (**Supplemental Figure 2**). Thus, the exacerbation of the Lyn^-/-^ phenotype with age, that occurred as a result of GILZ deficiency, was likely due to factors other than an age-related effect.

### GILZ Deficiency Worsened Glomerulonephritis in Lyn-Deficient Mice

**Figure 2:**
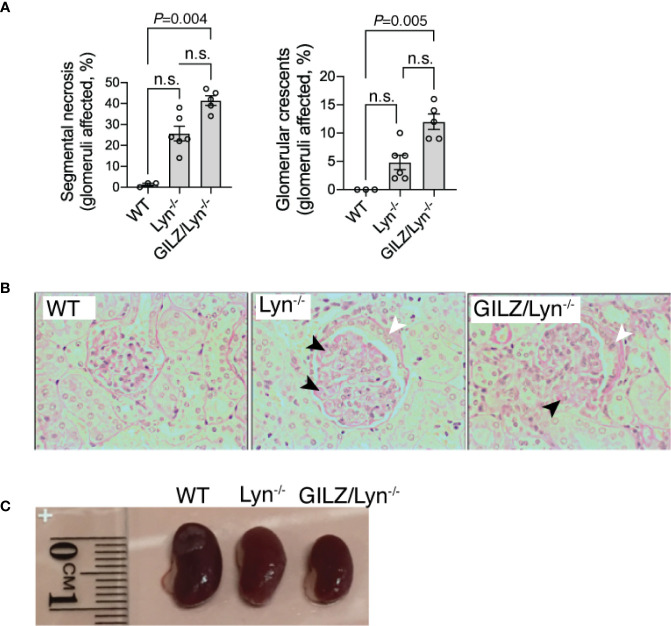
Effect of GILZ deficiency on glomerulonephritis in Lyn-deficient mice. **(A)** Renal injury was assessed by measurement of segmental necrosis (glomeruli affected in %) and glomerular crescents (glomeruli affected in %) in mice older than 200 days of age. **(B)** Representative images of glomerular lesions stained with periodic acid-Schiff (PAS) stain are shown, with segmental necrosis indicated with black arrowheads and glomerular crescents with white arrowheads. **(C)** Indicative differences in kidney size between mouse strains. Statistical significance was determined using Kruskal-Wallis test with Dunn’s correction for multiple comparisons (n=5-8). All data shown are mean ± SEM. NS, not significant.

Morbidity in the Lyn^-/-^ model is associated with the progressive development of renal injury. To determine GILZ whether deficiency worsens glomerulonephritis in the Lyn^-/-^ model, we compared the development of glomerulonephritis in Lyn^-/-^ mice with that in mice lacking both Lyn and GILZ (**Figure 2**). We observed that aged GILZ/Lyn^-/-^ mice had more severe glomerulonephritis compared to Lyn-deficient mice, featuring significantly increased segmental necrosis and glomerular crescents on PAS staining of kidney sections (**Figure 2A** with representative images shown in **Figure 2B**), and reduced kidney size (**Figure 2C**). Therefore, kidney damage in Lyn^-/-^ mice was exacerbated by GILZ deficiency.

### GILZ Deficiency Effects on Serum Autoantibodies in Lyn-Deficient Mice

**Figure 3:**
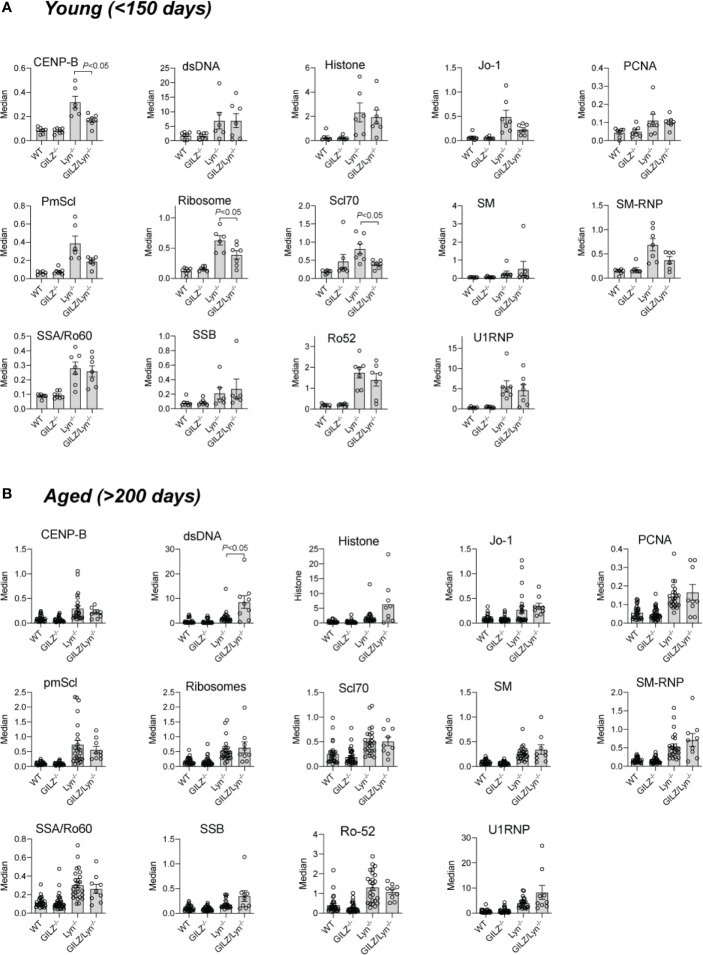
Autoantibodies reactive to specified extractable nuclear antigens (ENA) in mouse sera. Detection of IgG F(ab)2 autoantibodies against extractable nuclear antigens (ENA) detected by flow cytometry of serum from **(A)** young mice (<150 days) or **(B)** older (>200 days). Bars show median fluorescence intensity ± SEM normalized to positive control ENA beads. *P-*values were derived from Student’s *t*-test comparing Lyn^-/-^ with GILZ/Lyn^-/-^ mice. All data are presented as mean ± SEM (n = 6-24).

We next explored potential explanations for the heightened autoimmune phenotype observed in Lyn-deficient mice when GILZ was also deficient. The hyperactive B cells in Lyn*^-/-^* mice (20) and loss of B cell quiescence and tolerance we previously reported in GILZ^-/-^ mice (12) each result in the development of lupus-like autoimmunity. As a result, both strains produce ANA, and antibodies to ENA (dsDNA, histone, Smith (SM) and U1RNP) (12, 18). We sought to determine whether GILZ deficiency worsened the lupus phenotype of Lyn^-/-^ mice *via* exacerbation of autoantibody-mediated autoimmunity. While both Lyn^-/-^ and GILZ/Lyn^-/-^ mice expressed autoantibodies at a young age (**Figure 3A**), anti-dsDNA antibodies were present at significantly higher concentrations in the aged GILZ/Lyn^-/-^ mice compared to Lyn^-/-^ mice (>200 days) (**Figure 3B**); no other autoantibodies were significantly affected in aged GILZ/Lyn^-/-^ mice. Thus, while GILZ deficiency did not alter overall serum autoantibody production in Lyn^-/-^ mice in this study, it did significantly impact on an autoantibody specifically associated with nephritis in human SLE.

### Loss of GILZ Did Not Alter Immune Complex Deposition in Kidneys of Lyn-Deficient Mice

**Figure 4:**
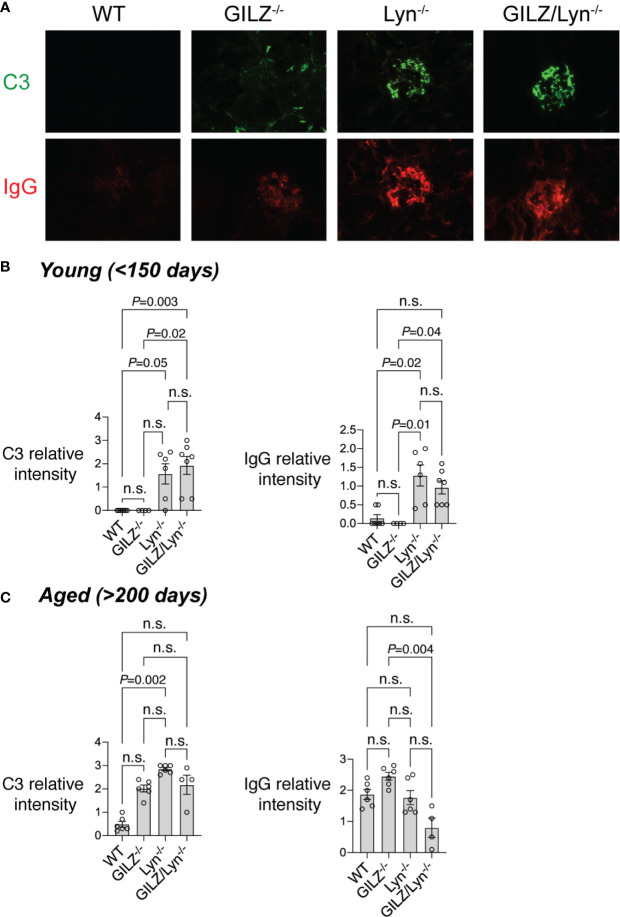
Immunofluorescence staining of immune complex deposition in glomeruli. **(A)** Immune complex (IC) deposition in glomeruli measured by immunofluorescence (IF) staining of complement component C3 (green) and immunoglobulin G (IgG; red). Representative images are shown from mice around 100 days of age. **(B)** IC deposition in mice young than 150 days or **(C)** older than 200 days was scored by a blinded expert using a scale of 0–3. (n=4–7). Statistical significance was determined using Kruskal-Wallis test with Dunn’s correction for multiple comparisons. All data are mean ± SEM. NS, not significant.

Previous studies report that Lyn-deficient mice develop glomerular immune complex deposition as early as 6–8 weeks of age and cumulative damage to kidneys subsequently worsens with ageing (18, 31). We previously reported that GILZ^-/-^ mice also develop mild immune complex-mediated glomerulonephritis as they age (12). In keeping with the generally equivalent levels of autoantibodies in Lyn^-/-^ and GILZ/Lyn^-/-^ mice, but in contrast to the higher levels of nephritis-associated anti-dsDNA antibodies, we detected negligible differences in glomerular immune complex deposition between mice of these two strains. While young mice (< 150 days old) of both Lyn^-/-^ and GILZ/Lyn^-/-^ strains showed deposition of C3 and IgG in the glomeruli, there was no significant difference in C3 deposition between the two genotypes (**Figure 4A** and quantified in **Figure 4B**). In aged mice, immunofluorescence staining of kidneys for C3 and IgG showed immune complex deposition in both Lyn^-/-^ and GILZ/Lyn^-/-^ mice, with no significant difference in the extent of C3 deposition observed (**Figure 4C**). Rather, there was a trend toward reduced IgG staining in glomeruli of aged GILZ/Lyn^-/-^ mice (**Figure 4C**). These observations suggest that GILZ deficiency worsened autoimmune disease outcomes in the Lyn^-/-^ model *via* processes other than through effects on the degree of autoantibody immune complex deposition in kidneys.

### GILZ Deficiency Affects Serum Cytokine Levels in Lyn-Deficient Mice

**Figure 5:**
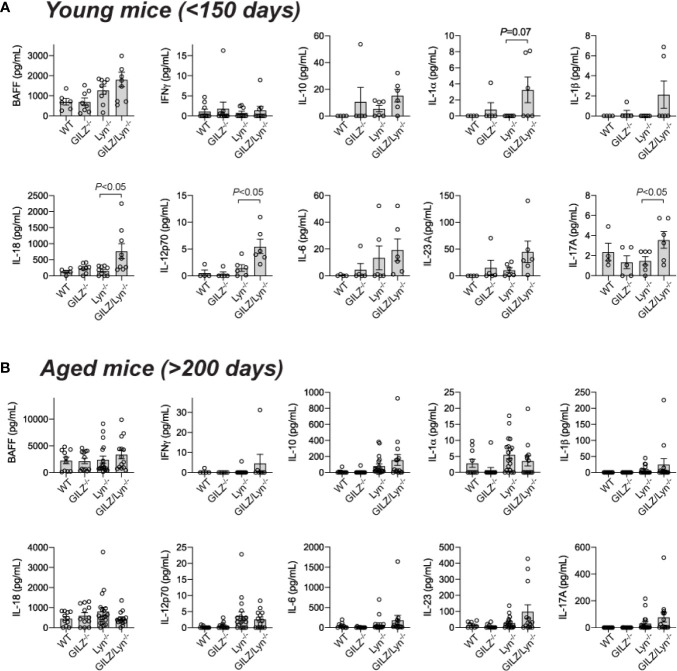
Effect of GILZ deficiency on serum cytokine expression in Lyn-deficient mice. Measurement of serum cytokines (pg/mL) including BAFF, IFNγ, IL-10, IL-1α, IL-1β, IL-18, IL-12p70, IL-6, IL-23A and IL-17A by Luminex in **(A)** young (< 200 days), and **(B)** aged mice (> 200 days). *P-*values were derived from Student’s *t*-test comparing Lyn^-/-^ with GILZ/Lyn^-/-^ mice. All data are presented as mean ± SEM (n = 4-20).

To further investigate the basis of the exacerbation of organ damage in GILZ/Lyn^-/-^ mice, we tested whether GILZ deficiency altered cytokine production in Lyn^-/-^ mice. Multiple cytokines were elevated in serum of young GILZ/Lyn^-/-^ mice, with substantial variance between individual mice as observed in human SLE. BAFF, IFNγ, IL-10, IL-1α, IL-1β, IL-6 and IL-23A all trended non-significantly numerically higher in young GILZ/Lyn^-/-^ mice than in Lyn^-/-^ littermate controls, and elevations in IL-18, IL-12 and IL-17A were significant (**Figure 5A**). In aged mice, in which the autoimmune phenotype was well established, these early significant differences were all attenuated (**Figure 5B**).

### GILZ Correlates With Cytokine Expression in Patients With SLE

**Figure 6:**
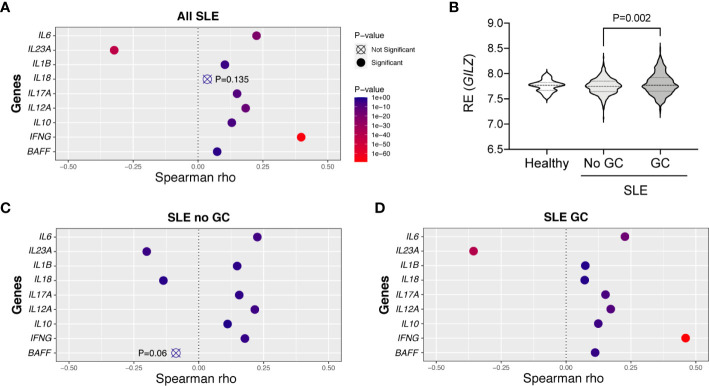
Correlations between GILZ and cytokine mRNA expression in patients with SLE. (Panels **A**, **C**, **D**) Publicly available microarray data was obtained from PBMCs of healthy controls and patients with SLE enrolled in the tabalumab trial at baseline (GSE88884). Relative mRNA expression levels of *IFNG, IL12A*, *IL6*, *IL1B*, *IL10*, *IL17A, IL23A*, *BAFF* and *IL18* were mined from the dataset and their correlations with GILZ were determined. Panels depict the degree to which GILZ expression is correlated with individual cytokines based on Spearman rho values, including all SLE patients (n=1,760; panel **A**), or limited to patients not taking glucocorticoids (GC; n=460, panel **C**), or patients taking GC (n=1293 included, panel **D**). **(B)** Distributions of the relative expression (RE) of GILZ isoform 1 in the healthy subjects (n = 60) and SLE patients divided into those not taking (n = 460), and those taking (n = 1293), glucocorticoids. The *P*-value was derived by Kruskal-Wallis test followed by Dunn’s correction for multiple comparisons.

To assess whether low GILZ expression is permissive of cytokine expression in human SLE, we mined the dataset GSE88884, which contains microarray data from PBMC from n =1,760 SLE patients. It is important to note that this study was limited to mRNA levels in PBMC rather than absolute amounts of circulating cytokines. Relationships with *GILZ* expression were strongest with *IL23A*, where a negative correlation was present in SLE patients (**Figure 6A**).

GILZ is a glucocorticoid-induced gene, and we assessed whether GILZ expression and its associations with cytokines were altered by glucocorticoid exposure in SLE patients. Healthy subjects (n = 60) and SLE patients not taking glucocorticoids (n = 460) had normally distributed and similar levels of expression of GILZ, with median, mean (± standard deviation) of 7.769, 7.727 (± 0.113) and 7.750, 7.749 (± 0.156) respectively. GILZ expression in PBMC from SLE patients taking glucocorticoids (n = 1293) was median and mean of 7.771 and 7.795 (± 0.211), and was significantly higher than in patients not taking glucocorticoids (*P*=0.002; **Figure 6B**). Since increased *GILZ* expression by glucocorticoids treatment could potentially obscure associations, we analyzed the subset of 460 patients not taking glucocorticoids. The level of *IL23A* was moderately negatively correlated to the expression of *GILZ* in both subsets of patients taking and not taking glucocorticoids (**Figures 6C, D**). Other cytokines including *BAFF* and *IL18* were negatively correlated with GILZ expression in non-glucocorticoid-using patients, although the relationship between *GILZ* and *BAFF* did not reach significance (with *P* = 0.06) (**Figure 6C**).

## Discussion

The treatment of autoimmune diseases like SLE with GC results in beneficial suppression of autoimmune inflammation, but also exposes patients to major adverse effects. The discovery of targets for the potential generation of safer GC mimics is an urgent unmet need. GILZ has been identified as a potential target for such an approach. Here, we investigated the effects of deficiency of GILZ on morbidity and mortality in Lyn^-/-^ mice, to glean insights into the pathogenic pathways that are regulated by GILZ in SLE.

GILZ is highly expressed in both mouse and human lymphoid and myeloid subsets, and is particularly abundant in macrophages (36–38), and low in plasmacytoid DCs, the key source of type I IFN in SLE (39). GILZ restrains DC maturation and activation, inducing DC-mediated promotion of antigen-specific Treg development (40–42). GILZ is highly expressed in macrophages, with clear anti-inflammatory effects (38, 43, 44). Moreover, down-regulation of GILZ occurs in acute inflammatory models, such as the caecal ligation and puncture (CLP) model of sepsis, mirroring the situation in patients with sepsis, although restoration of GILZ was protective against bacteremia and systemic inflammation (45–47).

Both Lyn-deficient and GILZ-deficient mice spontaneously develop lupus-like autoimmunity, and SLE patients have lower Lyn and GILZ expression (12, 48, 49). One study showed that by 25 weeks of age, 42% of Lyn^-/-^ mice were either developing autoimmune disease or had succumbed (18). In SLE patients, severe autoimmune disease with multi-organ damage results in marked loss of life expectancy when compared to the general population (50).

Here we report that loss of GILZ resulted in earlier onset and worsened splenomegaly in Lyn-deficient mice, suggesting a non-redundant role for GILZ in modulating the severity of autoimmune disease in this model. Investigations of GILZ deficiency are restricted to male mice, since the GILZ gene is on the X chromosome and its deficiency renders male mice sterile, thus knockout females cannot be bred, although male mice in the strains used here develop lupus-like autoimmunity in a relatively consistent manner over time. Since whole body knockout mice were employed, our data do not allow us to conclude which cell types are responsible for the phenotype we observed. However, several organ systems were affected by GILZ deficiency in Lyn^-/-^ mice.

Splenomegaly is an uncommon clinical manifestation of SLE that occurs particularly during active disease (30), the exact mechanism of which is not clearly understood, although it occurs almost universally in Lyn^-/-^ mice. Splenomegaly is known to be an active process that occurs due to splenic vessel inflammation, lymphoid hyperplasia (51), extramedullary hematopoiesis due to increased production of myeloid growth factors (52, 53) and in Lyn-deficient mice, due to accumulation of plasma cells, and Mac-1^+^ lymphoblasts, as early as 12 weeks (31). It has been demonstrated that splenomegaly is an IL-6-dependent phenotype in Lyn-deficient mice (20), however increases in IL-6 in Lyn^-/-^ mice were not further exacerbated in the absence of GILZ and mRNA expression of GILZ and IL-6 were significantly positively correlated in human SLE.

Lupus nephritis is a clinical phenotype that arises in 40–50% of SLE patients, and is associated with marked increases in morbidity and mortality. It is strongly associated with the presence of serum anti-dsDNA antibodies, and a recent phase III clinical trial has shown that outcomes are improved when neutralization of BAFF is added to glucocorticoids (54). Here, we confirmed the presence of immune complex-mediated nephritis in Lyn^-/-^ mice, and observed an exacerbation of renal damage in the absence of GILZ. In the present study, GILZ^-/-^ mice showed little evidence of splenomegaly, ENA, IgG or C3 in glomeruli of younger mice, or inflammatory cytokine expression, and for these reasons we did not assess the kidneys of GILZ^-/-^ for glomerular injury. However, GILZ deficiency worsened the severity of the Lyn^-/-^ phenotype, including glomerulonephritis. This suggests that GILZ induction may represent a mechanism through which GC act in the treatment of lupus nephritis in humans, although this remains to be proven.

In studying the processes that contribute to the increased severity of lupus-like disease upon loss of both GILZ and Lyn, we found that while autoantibody production overall was not significantly altered by the loss of GILZ, anti-dsDNA antibodies that are strongly associated with nephritis in human SLE were significantly increased in older double-deficient mouse strain compared to Lyn^-/-^. Despite this, glomerular immune complex deposition was not detectably increased in the absence of GILZ, suggesting that the effect of dysregulated BCR signaling in the absence of Lyn is a strong enough lesion in B cells that the absence of GILZ does not further exacerbate it and that effects on immune complex deposition do not explain worsened nephritis in the absence of GILZ.

Although SLE is an autoimmune disease characterized by hyperactive B cells, other cells including T cells and DC, and various cytokines, also play critical roles in SLE pathogenesis. SLE patients express increased levels of cytokines as products of innate and adaptive immune responses, and increased cytokine secretion is likely to drive tissue injury and end-organ damage in SLE. Interestingly, we found that GILZ deficiency permitted the early expression of an array of pro-inflammatory cytokines in young Lyn-deficient mice. The regulation of IL-17A and the possible regulation of the Th17-promoting cytokine IL-23A by GILZ in our study resonates with several previous reports including our own. We previously showed that GILZ inhibits IL-17A production and GILZ-deficient CD4 T cells produced increased IL-17 (21). Similarly, in acute kidney injury, GILZ was markedly suppressed by inflammation, and treatment with exogenous GILZ resulted in decreased IL-17, increased regulatory T cells (Treg) and IL-10, and prevention of cell death, demonstrating the renoprotective role of T cell GILZ (9). Indeed, a negative correlation was reported between GILZ mRNA and IL-17A levels in SLE patients (13, 14). Thus, our findings support the conclusion of these reports that GILZ is a regulator of the IL-17 axis, but suggest this effect has ramifications for organ damage in SLE.

While our study of gene expression levels in PBMC from SLE patients did not completely replicate our findings in the GILZ-deficient and GILZ-Lyn double-deficient mice, GILZ was robustly negatively correlated with expression of *IL23A*, an important Th17 driver, and with *BAFF* and *IL18* when studied in the subset of glucocorticoid-free patients. The role of BAFF has been comprehensively studied in SLE and neutralization of BAFF is approved for treatment of pediatric and adult SLE. Inhibition of BAFF is therapeutic in human lupus nephritis (54), and BAFF blockade in a phase III clinical trial of over 400 patients with SLE allowed glucocorticoid dose reduction (55). Thus, BAFF inhibition may occur downstream of glucocorticoid treatment, potentially mediated by GILZ.

The involvement of inflammasome activation, whence active IL-18 is derived, is comparatively understudied in SLE, although reports of associations of IL-18 in SLE are emerging. In two separate Asian populations, IL-18 predicted active renal SLE (56, 57), and an *IL18* gene polymorphism was identified to confer risk to renal involvement in SLE (57). A cross-sectional study of 28 SLE patients reported that IL-18 positively correlated with SLE disease activity, flares and anti-dsDNA expression (58). This same study identified that *IL23A* mRNA was strongly over-expressed in blood from patients with SLE (58). We recently showed, in the largest study to date, that serum IL-18 was elevated in SLE compared to healthy subjects, and was significantly associated with the presence of nephritis (59). Interestingly, glucocorticoid treatment returned elevated IL-18 to control levels in a group of 30 previously untreated SLE patients (60); our data suggest that GC-induced GILZ may be at play in this response.

In conclusion, our findings demonstrate that any additional effect of GILZ on regulation of spontaneous B cell activation, which we previously described, is modest in the context of autoimmune disease development in the Lyn^-/-^ mouse model. In contrast, GILZ appears to modulate cytokine-dependent inflammation in SLE, resulting in exacerbation of renal injury. This supports the hypothesis that GILZ supplementation could be beneficial in SLE, a concept that merits further investigation, such as through the use of GILZ transgenic animal models.

## Data Availability Statement

The original contributions presented in the study are included in the article/**Supplementary Material**. Further inquiries can be directed to the corresponding author.

## Ethics Statement

The animal study was reviewed and approved by Monash Animal Ethics Committee.

## Author Contributions

CN, WD, and JF planned and conducted experiments, analyzed data, and helped to prepare the manuscript. JL, WZ, FV, LG, JO, MP, MC, RS, and MH conducted experiments and/or analyzed data. JH, EM, and SJ planned experiments, analyzed data and prepared the manuscript. All authors contributed to the article and approved the submitted version.

## Funding

This work was supported by the Lupus Research Alliance as part of the Distinguished Innovator Award to EM.

## Conflict of Interest

The authors declare that the research was conducted in the absence of any commercial or financial relationships that could be construed as a potential conflict of interest.

## Supplementary Material

Supplementary Figure 1 Health summary from sentinel animals housed in the same room as the strains used in this study over the period 2013-2020. Only organisms that were detected are shown in this figure. The degree of coloring corresponds to the proportion of sampled animals that tested positive.

Supplementary Figure 2 GILZ expression in healthy subjects in publicly available datasets **(A)** GSE123698 and **(B)** GSE69832 (26, 27). **(C)** GILZ expression in (n = 1,760) patients with SLE, determined by extracting the data for the probes TC0X001262.hg.1:1, TC0X001262.hg.1:2 and TC0X001262.hg.1:3, which identified GILZ, from the public dataset GSE88884 and averaging the three probe set values for each subject (28, 29).
