# Supplementary figures and images for: GILZ Regulates the Expression of Pro-Inflammatory Cytokines and Protects Against End-Organ Damage in a Model of Lupus
Source: Front Immunol. 2021 Apr 6;12:652800. doi: 10.3389/fimmu.2021.652800 (PMC8056982; doi:10.3389/fimmu.2021.652800)

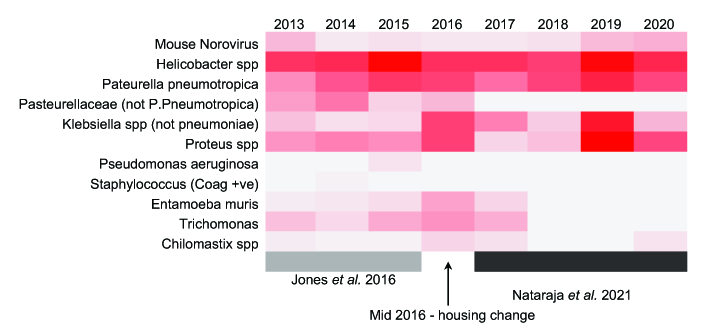

Supplement: Supplementary Figure 1 — Health summary from sentinel animals housed in the same room as the strains used in this study over the period 2013-2020. Only organisms that were detected are shown in this figure. The degree of coloring corresponds to the proportion of sampled animals that tested positive. [file Image_1.tif]

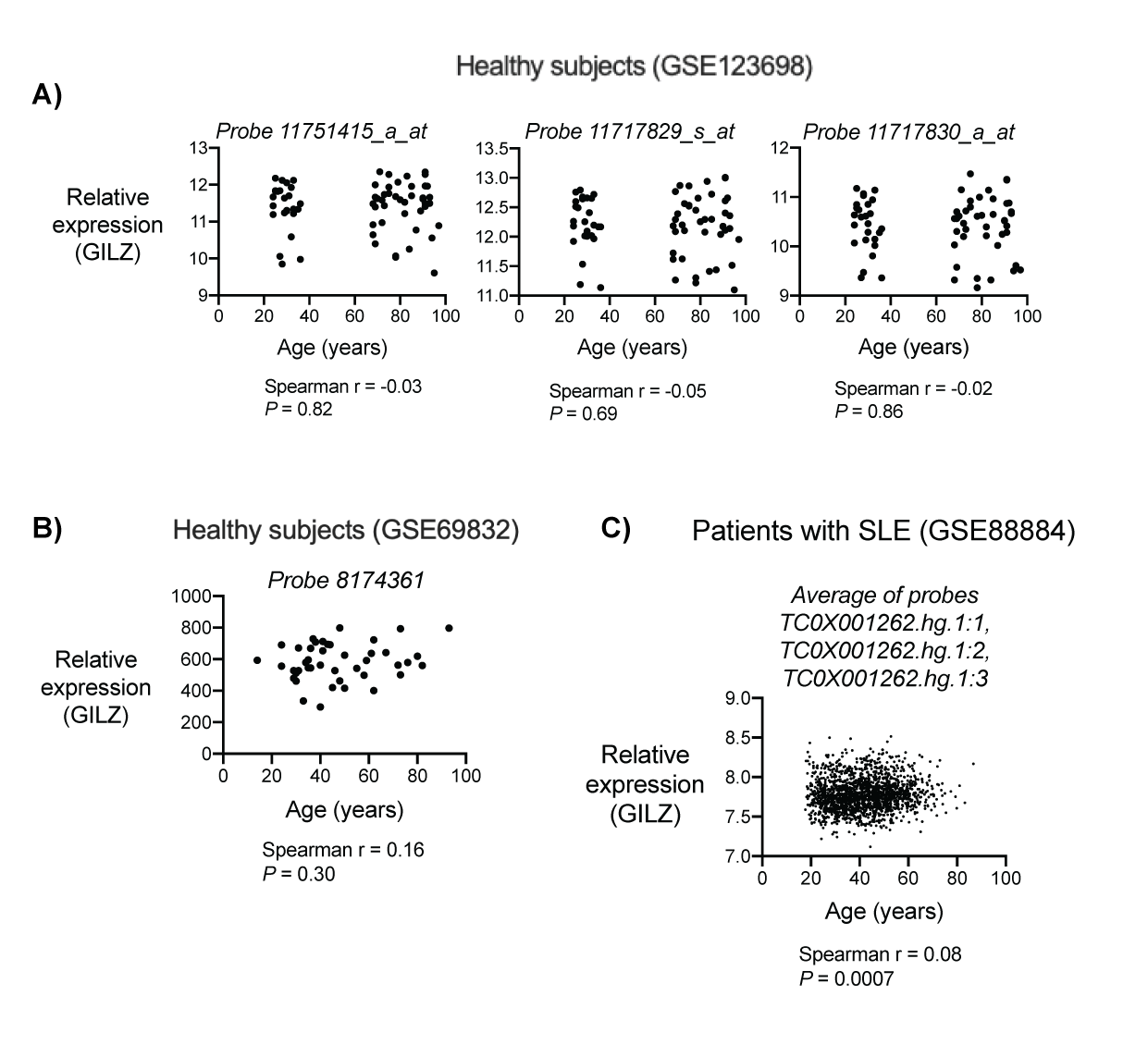

Supplement: Supplementary Figure 2 — GILZ expression in healthy subjects in publicly available datasets (A) GSE123698 and (B) GSE69832 (26, 27). (C) GILZ expression in (n = 1,760) patients with SLE, determined by extracting the data for the probes TC0X001262.hg.1:1, TC0X001262.hg.1:2 and TC0X001262.hg.1:3, which identified GILZ, from the public dataset GSE88884 and averaging the three probe set values for each subject (28, 29). [file Image_2.tif]
